# How to assess visual function in acquired brain injury—Asking is not enough

**DOI:** 10.1002/brb3.1958

**Published:** 2020-11-23

**Authors:** Märta Berthold‐Lindstedt, Jan Johansson, Jan Ygge, Kristian Borg

**Affiliations:** ^1^ Division of Rehabilitation Medicine Department of Clinical Science Karolinska Institute Danderyd University Hospital Stockholm Sweden; ^2^ Eye and Vision Department of Clinical Neuroscience Karolinska Institute Stockholm Sweden

**Keywords:** brain injuries, ocular motility disorders, stroke, traumatic brain injury, vision disorders

## Abstract

**Background:**

Acquired brain injury affects many brain areas and causes a range of dysfunctions including vision‐related issues. These issues can have negative impacts on rehabilitation progress and activities of daily life but may easily be overlooked. There is no common recommendation about how to assess visual impairments after ABI. The purpose of this study was to estimate the frequency of objectively measures oculomotor dysfunctions, and also how these findings are related to two inventories intended to support detection of visual impairment.

**Methods:**

The study was cross‐sectional and included 73 outpatients. In addition to the standard evaluation program, the patients went through a comprehensive optometric examination. The inventories used were the Vision Interview (VI) and the Convergence Insufficiency Symptom Survey (CISS).

**Results:**

All three types of examinations showed a high proportion vision‐related symptoms. Fusion vergence was the most common objectively measured finding, 83%. There were seven statistically significant associations between five VI items and five visual deficits. The strength of associations was moderate (Phi 0.261–0.487, *p* < .05). The sensitivity and specificity of the CISS were moderate.

**Conclusion:**

We found high percentages of the patients with visual symptoms and dysfunctions. Due to the complexity of visual symptoms and functional deficits in ABI, we find it necessary to combine both symptom assessment and vision examination in order to capture visual function issues.

## Graphical abstract

The study explores visual dysfunctions after acquired brain injury in a neurorehabilitation setting and studies the association with visual symptoms. We conclude that both symptom assessment and objective measurements are required to capture these issues.

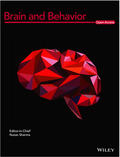

## 1 INTRODUCTION

Visual dysfunctions are common after acquired brain injury (ABI) with an occurrence of 50%–70% and may affect visual acuity, visual field, and oculomotor functions (Ciuffreda et al., 2007; Greenwald et al., 2012; Rowe, 2016). These functions constitute an important base in the hierarchy of visual processing (Warren, 1993). Interference of these functions may aggravate tasks that require efficient processing, for example, reading, mobility, and many daily activities (Kerkhoff, 2000; Simons, 1993). Unattended, these problems can negatively affect the ability to perform rehabilitation activities, to take part in activities of daily life (Ciuffreda et al., 2007; Heitger et al., 2006) and thereby interfere with life quality and satisfaction.

There is no standard recommendation of assessment of visual impairments after ABI. Dysfunctions that are fairly apparent, like visual field defects, manifest strabismus, restricted eye motility or patient reported double vision, tend to be appropriately referred for diagnosis and treatment. Less obvious oculomotor problems risk being overlooked since they are difficult to distinguish in an overall complex constellation of symptoms. Another issue is that the patient may not be aware of, or relate the symptoms to visual dysfunctions (Berthold‐Lindstedt et al., 2017). If symptoms of oculomotor dysfunctions are found, there are concrete ways of addressing these with compensatory and/or restoring interventions (Conrad et al., 2017; Rowe et al., 2019; Simpson‐Jones & Hunt, 2019). It has therefore been suggested that the use of a structured interview is helpful. In a former study where we used the Visual Interview (VI), we found more than 50% with vision‐related symptoms (Berthold‐Lindstedt et al., 2017).

The purpose of the current study was threefold.

1. To estimate the frequency and type of visual dysfunctions, objectively measured, in a common patient group in a neuro‐rehabilitation setting. The measured visual functions were visual acuity, visual field, eye focusing (accommodation), and eye alignment (heterophoria, convergence, fusional vergences).
2. To evaluate the association between symptoms found in the VI and objectively measured visual dysfunctions.
3. To evaluate specific near work related symptoms using Convergence Insufficiency Symptom Survey (CISS) and its sensitivity and specificity for detecting clinical signs indicating visual dysfunction.

## 2 METHODS

The study was cross‐sectional and aimed at including all ABI patients qualifying for an ABI outpatient rehabilitation program. All patients (*n* = 73) had suffered a moderate to severe ABI with persistent disability corresponding to grade 4–7 on the Glasgow Outcome Scale Extended (GOSE) (Teasdale et al., 1998) (Table 1). The diagnoses included stroke, traumatic brain injury (TBI), subarachnoid hemorrhage (SAH) infection, tumor, hypoxia, and other. Other diagnoses included arteriovenous malformation (*n* = 1), hydrocephalus (*n* = 2), and idiopatic intracranial hypertension (*n* = 1). The patients had been referred from caregivers in the Stockholm area for evaluation of rehabilitation needs. To qualify for a rehabilitation program, the patient had to be medically stable and in need for rehabilitation based on the standard evaluation program. The evaluation included medical, cognitive and psychological status as well as activity/occupational limitations. The exclusion criteria for the study were cognitive disabilities not due to the current brain injury, ongoing drug abuse, or extensive aphasia. Upon admittance to the rehabilitation program, the patient was informed verbally and in writing concerning the study. If accepting to participate, the patient was asked to give written informed consent. The study adhered to the tenets of the Declaration of Helsinki and was approved by the regional ethics board (Dnr 2016/408‐32).

**TABLE 1:** Demographics

	All	Stroke	TBI	Infection	Hypoxia	Tumor	SAH	Other
	*n* = 73	*n* = 32	*n* = 12	*n* = 9	*n* = 6	*n* = 6	*n* = 4	*n* = 4
Male/ Female	42/31	22/10	8/4	2/7	5/1	3/3	1/3	1/3
Median age, years (min–max)	50 (20–64)	51 (29–63)	35 (21–50)	50 (27–57)	53.5 (20–64)	56 (46–63)	54 (38–59)	40 (25–58)
Time since injury								
0–3 months	14	8	5				1	
4–6 months	21	11	4	2	2	1	1	
7–12 months	20	9	1	3	3	3		1
13–24 months	10	3	2	1	1	1	1	1
>24 months	8	1		3		1	1	2
Glasgow outcome scale extended								
GOSE 4	6	3	1	1	1			
GOSE 5	40	20	8		4	3	3	2
GOSE 6	25	9	3	8	1	3	1	
GOSE 7	2							2

Note

GOSE 4: Upper severe disability; GOSE 5: Low moderate disability; GOSE 6: Upper moderate disability; GOSE 7: Low good recovery.

The visual function examination was carried out by a licensed optometrist (JJ) within two weeks of the admittance. The optometrist was given background information regarding the type and time of injury but was restricted from the results of the VI. The visual function assessment followed a study protocol including visual acuity, refractive error, eye alignment (covertest, fusional vergences), near visual function (near point and facility of convergence and accommodation), stereo vision, and eye motility. Accommodative functions were only measured in patients below 40 years due to the physiological deterioration of these functions with age.

Visual dysfunctions were diagnosed according to criteria derived from the literature (Antona et al., 2008; Cacho‐Martinez et al., 2014; Gall et al., 1998; Garcia et al., 2000; Goss & Becker, 2011; Momeni‐Moghaddam et al., 2014; Pellizzer & Siderov, 1998; Yekta et al., 2017) (Table 2).

**TABLE 2:** Visual function, impairment, diagnostic criteria, and typically associated symptoms

Visual function	Type of impairment	Criteria	Typical symptoms
Visual acuity	Uncorrected refractive error, amblyopia, damaged visual pathways	Monocular visual acuity below decimal 1.0	Manifest or intermittent blurred vision at distance
Peripheral visual field	Partial or complete loss of peripheral vision due to damaged visual pathways	As determined with standard visual field testing at the ophthalmologist's office, mainly via Humphrey Visual Field Analyzer	Difficulties with visual overview and ambulation
Accommodation	Defective amplitude (near point)	Accommodative amplitude (D) less than minimum expected according to the Hofstetter formula (15−1/4 age).	Difficulty to achieve and maintain focus at near, eye strain.
	Infacility	<4.5 cpm with age‐ appropriate lens power (±1 D to ±2 D lens flipper).	Delayed clarity of vision when altering focus between near and far.
Convergence	Defective near point	Near point >10 cm	Intermittent double vision at near, eye strain, headache.
	Infacility	<11 cpm with 3 pd BI/ 12 pd BU prism flipper (prepresbyope, age < 40) <7 cpm with 3 pd BI/ 12 pd BU prism flipper (presbyope, age ≥ 40)	Delayed clarity of vision when altering focus between near and far.
Fusional vergence	Below minimum expected amplitudes for break point for either NFV or PFV	NFV at far: minimum 6 pd BI PFV at far: minimum 13 pd BO NFV at near: minimum 13 pd BI PFV at near: minimum 19 pd BO	Intermittently blurred or double vision, floating words, apparent movement of fixated object, strenuous to maintain eye contact, eye strain, headache.

Abbreviations: BI, Bas In; BO, Base Out; cpm, cycles per minute; D, diopter; NFV, negative (divergent) fusional vergence; pd, prism diopter; PFV, positive (convergent) fusional vergence.

The VI was administered by a physician at the admission of the patient to the day‐care program according to the procedures earlier described. The VI consists of 20 items to which the patients respond with a yes or no, (Table 4). It holds two general questions; if you have experienced vision change after illness/injury or if you have had an eye examination. Seventeen questions are about different symptom on functional or activity level, Table 4. The final item is a 10‐step scale where the patient is asked to grade the impact of visual function issues on daily activities where 0 equals no impact and 10 the worst possible impact.

CISS is a survey that focuses on symptoms related to near work such as reading or computer work. The CISS is originally intended for the detection of convergence insufficiency (Borsting et al., 2003; Rouse et al., 2004) where the diagnostic criteria consist of combined clinical signs concerning heterophoria, near point of convergence, and fusional vergences (Rouse et al., 2004) but have also been used in studies of binocular vision issues in patients with mild traumatic brain injury (Capo‐Aponte et al., 2017; Thiagarajan et al., 2014). The CISS was filled in by the patient in conjunction with the vision examination. The survey consists of 15 symptom items related to reading and near work. The patient grades each item on scale: never (0), rarely (1), sometimes (2), often (3), or always (4). A total score of 21 or more can be considered the cutoff between normal and abnormal levels of symptoms (Rouse et al., 2004).

### 2.1 Statistical analyses

Analysis of results was performed with IBM SPSS Statistics 26. Distribution tests were performed with chi‐square or Fisher's exact. The visual interview consists of items to which the patient responds with a yes or no and thus treated as dichotomous values. The findings in the vision examination were also treated as dichotomous values. Continuous measures of visual function (vergence, accommodation) were converted to a dichotomous value based on the diagnostic criteria in Table 2.

## 3 RESULTS

A total of 73 patients were included in the analysis. Six patients, out of the 79 patients who had been recruited initially, were excluded: two due to discontinued rehabilitation program and four due to incomplete data.

### 3.1 Visual examination

The findings of oculomotor clinical signs are described in Table 3. The visual examination found subnormal visual acuity in 19 patients (26.4%). The reasons were uncorrected or insufficiently corrected refractive error (*n* = 12), ocular health issues (*n* = 3), amblyopia (*n* = 3), and damage to the visual pathways associated with the ABI (*n* = 1). Visual field defects were found in 15 patients (20.8%) which included homonymous hemianopia (*n* = 7) or quadrant anopia (*n* = 5) and other (*n* = 2). Accommodative functions were measured in 22 patients, where five patients (22.7%) showed insufficient accommodation.

**TABLE 3:** Oculomotor findings in the visual examination

	All (*n* = 73)	Stroke (*n* = 32)	TBI (*n* = 12)	Infection (*n* = 9)	Hypoxia (*n* = 6)	Tumor (*n* = 6)	SAH (*n* = 4)	Other (*n* = 4)
Eye alignment issues								
Near point of convergence	18 25.0%	8 25.8%	4 33.3%	2 22.2%	0 0%	1 16.7%	1 25.0%	2 50.0%
Vergence facility	35 48.6%	14 45.2%	7 58.3%	4 44.4%	3 50.0%	3 50.0%	3 75.0%	1 25.0%
Fusion vergence	60 83.3%	25 80.6%	10 83.3%	8 88.9%	6 100.0%	3 50.0%	4 100.0%	4 100.0%
Strabism	2 2.8%	1 3.1%	1 8.3%					

### 3.2 The visual interview

A total of 65 patients (89.0%) reported at least one symptom. The most frequent symptoms were reading difficulties, a general vision concern, and difficulty to remember when reading (Table 4). The visual analog scale showed a median of 3.75 (min 0 max 10).

**TABLE 4:** The responses from the Visual interview presented in descending order

Item (Item no)	Number of responses	Percentage of patients
Reading difficulties (16)	47	64%
General vision concern (1)	44	60%
60%Difficulty remembering while reading (17)	39	53%
Hypersensitivity to glare (4)	31	42%
Blurry vision (7)	31	42%
Need more light while reading (6)	24	33%
Frequently bumping into people or objects (13)	24	33%
Difficulty with ambulation (12)	21	29%
Visual field affected (3)	19	26 %
Neck pain (11)	19	26%
Headache when reading (18)	19	26%
Difficulty with depth perception (14)	18	24%
Needing more light in general to see well (5)	17	23%
Other visual concern (10)	11	15%
Double vision (2)	10	14%
Problems with recognizing faces (9)	8	11%
Difficulty with eye‐hand coordination (15)	8	11%
Affected color perception (8)	4	5%

### 3.3 The convergence insufficiency symptom survey

The median CISS score across all diagnoses was 23 (min 1, max 49) with 39 patients (54.2%) scoring 21 or above. Within diagnoses, the share of patients who scored 21 or above spanned between 45.2% (stroke) and 75% (other).

### 3.4 Associations between VI and clinical findings

The first question of the VI concerns if the patient has experienced any general vision concern. A total of 45 patients admitted, while 25 patients denied any general vision concern. The findings in each group are described in Table 5.

**TABLE 5:** Clinical findings in patients reported versus denying general vision concern

	General vision concern "Yes" "No"	
	No of patients reporting (*n* = 45)	No of patients denying (*n* = 25)
Subnormal visual acuity	13 (28.9%)	6 (24.0%)
Visual field defect	12 (26.7%)	3 (12.0%)
Convergence issues	25 (55.6%)	14 (56.0%)
Fusional vergence issues	39 (86.7%)	18 (72.0%)
Accommodation issues	2 (4.4%)	3 (12.0%)

A chi‐square or Fisher's exact test for association was conducted between the VI item responses and the presence of visual dysfunctions. There were seven statistically significant associations (Table 6). The strength of the associations was moderate (Phi 0.261–0.487).

**TABLE 6:** Associations between symptoms identified with VI and visual dysfunctions

Item (Item No)	Vision deficit				
	Visual acuity	Positive fusional vergence at far	Positive fusional vergence at near	Vergence infacility	Visual field defect
General vision concern (1)		Chi‐square 5.228, *df* = 1, *p* = .022, Phi = 0.29	Chi‐square 10.397, *df* = 1, *p* = .001, Phi = 0.409		
Double vision (2)			Fisher Exact *p* = .005, Phi = 0.261		
Visual field affected (3)				Fisher Exact *p* = .01, Phi = 0.343	Fisher Exact *p* = .000, Phi = 0.487
Frequently bumping into people or objects (13)					Fisher Exact *p* = .027, Phi = 0.302

### 3.5 Symptoms according to CISS versus visual deficits

We examined the association between CISS score and separate clinical signs at near testing, that is, the usefulness of CISS score to detect deficiencies in near point of convergence, vergence facility, and positive fusional vergence. A cutoff score of 21 resulted in sensitivity 66.7%/ specificity 53.2% for near point of convergence, sensitivity 54.3%/ specificity 45.8% for vergence facility, and sensitivity 64.5%/ specificity 60.6% for positive fusional vergence. Finally, the Youden's index (Sensitivity + Specificity −1) was applied to find the CISS score that maximized sensitivity and specificity (Table 7).

**TABLE 7:** The CISS Score that maximizes sensitivity and specificity

Clinical sign	CISS Score cutoff	Sensitivity (%)	Specificity (%)	Youden's index
Near point of convergence	25	55.6	68.1	0.24
Vergence facility	17	65.7	45.8	0.12
Positive fusional vergence	21	64.5	60.6	0.25

For reference, we also evaluated the sensitivity and specificity of CISS when strictly applying the diagnosis criteria for convergence insufficiency (Rouse et al., 2004). It then resulted in a sensitivity of 71.4% and specificity of 46.6%. However, only 7 patients met the strict diagnostic criteria.

## 4 DISCUSSION

The aim of this study was to estimate the frequency of visual impairment and its associations with self‐reported visual symptoms. The patients were recruited consecutively with the intention to study a typical clinical population admitted at an outpatient neuro‐rehabilitation clinic. The distribution of diagnoses was in fair unanimity with our previous work (Berthold Lindstedt et al., 2019; Berthold‐Lindstedt et al., 2017) with stroke and TBI constituting approximately 60%.

A striking finding was that one quarter of the patients had subnormal visual acuity, and in a majority of these patients, it was due to uncorrected or insufficiently corrected refractive error. Adequate correction is the basis for any further vision interventions. It relieves eye strain associated with squinting or effort to overcome blurred vision. It also provides optically clear images in the eyes which is important for the sensory‐motor processing of the visual input.

The most common clinical signs were vergence‐related (eye alignment) issues followed by visual acuity and visual field loss. High rates of these impairments have also been shown previously (Ciuffreda et al., 2007; Rowe et al., 2009). Vergence eye movements, including convergence and fusional vergences, are relevant for the continuous adjustment of eye position to make the visual axes of the eyes point at objects of regard at different depth. Inadequate vergence eye movements cause problems since it impairs the capacity to maintain a stable and clear single vision. This may have implications for reading, computer work, and other task at near distance but also for ambulation, where the spatial awareness depends on efficient and perseverant continuous scanning of three‐dimensional visual space. Screening for convergence issues can be performed using a pen, but fusional vergences and vergence facility may be difficult to catch and an objective examination is therefore necessary.

The VI showed that a high proportion of the patients (89%) experienced visual symptoms. The most common were related to reading, a general vision concern, followed by hypersensitivity to glare and blurred vision. Twenty‐five patients (34%) denied visual problems although 18 of these had fusion vergence issues and 14 had convergence issues. This supports our previous findings that a generally held question to the patients about if they had experienced vision changes is not enough, one has to ask more specific questions (Berthold‐Lindstedt et al., 2017). A visual examination may serve as an important part of the mapping of functional deficits and also in increasing the self‐awareness of issues.

The sensitivity and specificity of the CISS regarding clinical signs were fairly low. It improved somewhat when applying strict diagnostic criteria for convergence insufficiency for which it was originally intended. Still it did not reach the same level as found in the original reports (Rouse et al., 2004). Based on clinical observations, we have two theories regarding this outcome. One is that this patient group may find it hard to relate fully to the questions in CISS. It may be due to difficulties to discern vision‐related issues from others, like issues related to cognition and fatigue. Another theory is that the patients have not yet resumed the activities in regard.

There were seven statistically significant associations between vision deficits and VI items. The item General vision concern in the VI was associated with deficits of fusional vergences. The concept of fusional vergences can be elusive unless clinically measured. However, the typical symptoms associated with reduced fusional vergences; intermittently blurred or double vision, floating words, apparent movement of fixated object, strenuous to maintain eye contact, eye strain, or headache may well fit the patient's notion that something, however, diffuse, is different with vision. It is known that vergence issues are associated with fatigue and illness (Rutstein & Daum, 1998). In an earlier published study, we found an association between fatigue and self‐reported vision symptoms. The high rate of fusion vergence in this study may be one of the reasons for this association (Berthold Lindstedt et al., 2019).

Double vision was associated with defects of fusional vergences at near. Intermittent double vision may appear when the fusional vergence fail to maintain eye alignment. Near work like reading, looking at a mobile phone, and computer work means increased demands of the visual system. Fusional vergence has an important role in maintaining the convergent position of the eyes during near work as well as compensation for any heterophoria which is very commonly occurring, especially at near viewing.

There was an association between visual field defects and vergence infacility, that is, an impaired ability to flexibly alter focus between near and far. There is an indirect relationship that may explain this. Visual field defects hamper fusional vergences which in turn hamper vergence flexibility. The finding corresponds to our clinical experience. We have noticed that it is better to start the vision rehabilitation of a vision field defect with exercises for stable vision, including vergence facility and fusional vergences, before beginning compensatory eye movement training.

All measurements showed a high rate of visual dysfunctions. This is in agreement with previous studies and other studies concerning frequencies of visual impairment and dysfunction after stroke or TBI (Berthold‐Lindstedt et al., 2017; Ciuffreda et al., 2007; Merezhinskaya et al., 2019; Rowe et al., 2009; Schuett et al., 2012) and indicate the need of a visual assessment and rehabilitation based on the patient's capacity of processing visual information.

Some of the visual functions that we have assessed can be fairly easy included in the examination performed by a physician. However, certain visual functions such as refractive error, visual field defects, and eye alignment (vergence) functions require an assessment by a vision specialist.

In the subjective questionnaire, the patient´s own experience of visual changes is revealed, which also covers problems with higher complexity in the hierarchy of the brain. Thus, both objective and subjective methods are necessary to create an image of the patient's visual dysfunction. The clinical observations made by the neuro‐rehabilitation team are then added, which gathered, provide an increased understanding of the patient´s more complex vision‐related problems on activity and participation level. With a better understanding of vision and its importance for the dynamic ability to interact with the environment, methods of rehabilitation after ABI may improve.

The competence to perform objective assessments is usually found outside the neuro‐rehabilitation units. The importance of integrating a vision specialist in the neuro‐rehabilitation team for further development of vision rehabilitation has been address in several articles (Roberts et al., 2016; Rowe et al., 2015), and the American Congress of Rehabilitation Medicine, ACMR, has described such a rehabilitation model in their article A conceptual model for vision rehabilitation (Roberts et al., 2016). Different roles of the vision specialists and the neuro‐rehabilitation team were described and how to interact and, thus, be able to develop vision assessment and rehabilitation after ABI. The last six years our team has been working in a similar way. This concept paves the way for research and development within an earlier neglected area and our hope is that such a model can be introduced more generally in rehabilitation after ABI.

### 4.1 Limitation

We have chosen to study a clinical population that we believe represents the clinical reality. There is a risk that the major diagnoses obscure the issues present in the smaller diagnosis groups. A specific study with, for example, only patients suffering from encephalitis might give another profile of visual impairments. However, our clinical experience shows a high level of vision problems in all the patients with different diagnoses included in our study.

Another aspect is the diagnosis criteria for visual dysfunctions. For example, the diagnosis criteria for fusion vergence dysfunction may differ.

## CONFLICT OF INTEREST

The authors have no conflicts of interest to declare.

## AUTHOR CONTRIBUTIONS

All authors took active part in the design and realization of the study with some specific responsibilities as described hereafter. MBL performed examinations, compiled and analyzed data, and drafted the manuscript. JJ performed vision examinations, compiled and analyzed data, and assisted in drafting the manuscript. JY contributed with visual function expertise and reviewed the manuscript. KB acted as project manager and reviewed the manuscript.

### Peer Review

The peer review history for this article is available at https://publons.com/publon/10.1002/brb3.1958.

## Data Availability

The data that support the findings of this study are available from the corresponding author upon reasonable request.
